# *NOTCH3* drives fatty acid oxidation and ferroptosis resistance in aggressive meningiomas

**DOI:** 10.1007/s11060-025-05208-5

**Published:** 2025-09-09

**Authors:** Nishanth S. Sadagopan, Mateo Gomez, Shashwat Tripathi, Leah K. Billingham, Susan L. DeLay, Martha A. Cady, Harrshavasan T. S. Congivaram, Tzu-Yi Chia, Hanxiao Wan, Si Wang, David R. Raleigh, Faith C. Kaluba, Evan C. Lien, Amy B. Heimberger, Catalina Lee-Chang, Mark W. Youngblood, Stephen T. Magill, Jason M. Miska

**Affiliations:** 1 Department of Neurological Surgery, Northwestern University Feinberg School of Medicine, Chicago, IL USA; 2 Feinberg School of Medicine, Robert H. Lurie Comprehensive Cancer Center, Lou and Jean Malnati Brain Tumor Institute, Northwestern University, Chicago, IL 60611 USA; 3 Department of Radiation Oncology, Neurological Surgery, and Pathology, University of California, San Francisco, CA USA; 4 Department of Metabolism and Nutritional Programming, Van Andel Institute, Grand Rapids, MI USA

**Keywords:** *NOTCH3*, Meningioma metabolism, Fatty acid oxidation, Ferroptosis, Tumor microenvironment

## Abstract

**Purpose:**

*NOTCH3* is increasingly implicated for its oncogenic role in many malignancies, including meningiomas. While prior work has linked *NOTCH3* expression to higher-grade meningiomas and treatment resistance, the metabolic phenotype of *NOTCH3* activation remains unexplored in meningioma.

**Methods:**

We performed single-cell RNA sequencing on NOTCH3 + human meningioma cell lines. Using the CH157-MN meningioma cell model, we overexpressed *NOTCH3* intracellular domain (ICD) and performed untargeted metabolomic, lipidomic, and bulk RNA sequencing analyses as well as functional metabolic assays.

**Results:**

We show that *NOTCH3* mediates a metabolic shift towards fatty acid oxidation (FAO), depleting lipid availability and conferring resistance to ferroptosis. Single-cell RNA sequencing revealed a correlation with CD36, a key fatty acid transporter. Furthermore, patient-derived primary meningioma lines stratified by *NOTCH3* expression confirmed higher *CD36* expression and increased maximal mitochondrial respiration in *NOTCH3*-high cells in the presence of palmitate, supporting enhanced FAO. *NOTCH3* ICD overexpression (OE) exhibited depletion of fatty acid pools, alongside transcriptional upregulation of canonical FAO genes. Functional mitochondrial assays confirmed elevated oxidative respiration in the presence of palmitate compared with controls. Additionally, *NOTCH3* OE cells exhibit increased resistance to RSL3-induced ferroptosis, a phenotype that was reversed with CPT1 inhibition.

**Conclusion:**

These data establish a link between *NOTCH3* signaling, lipid metabolic reprogramming, and ferroptosis evasion in aggressive meningioma cells. This metabolic shift may contribute to the malignant behavior observed in *NOTCH3 +* meningiomas, offering new insight into the biochemical vulnerabilities of these tumors.

**Supplementary Information:**

The online version contains supplementary material available at 10.1007/s11060-025-05208-5.

## Introduction

The *NOTCH* signaling pathway plays a fundamental role in cell fate determination, differentiation, and tissue homeostasis. Among the four receptors (*NOTCH1–4*), *NOTCH3* expression is more restricted and is predominantly found in vascular smooth muscle, the central nervous system, and thymocytes [1–3]. While deletions of *NOTCH1* and *NOTCH2* are embryonically lethal, *NOTCH3* deletion is not [4, 5]. The NOTCH3 transmembrane receptor undergoes a series of cleavage events, via ADAM10 metalloproteases and γ-secretase complex, yielding an extracellular domain (ECD) and an intracellular domain (ICD) [6, 7]. The active ICD is then translocated to the nucleus, where it participates in regulating gene transcription.

*NOTCH3* plays an important role in the survival of vascular smooth muscle cells, which explains its expression pattern in this tissue [8]. *NOTCH3* has been implicated in vascular pathologies, including cerebral autosomal dominant arteriopathy with subcortical infarcts and leukoencephalopathy (CADASIL) as well as multiple cancers, including T-cell lymphoma, breast cancer, pancreatic adenocarcinoma, and others [9–12]. In gliomas, *NOTCH3* overexpression has been associated with the promotion of glioma cell proliferation and therapeutic resistance, suggesting a conserved oncogenic role across diverse tumor types [13, 14].

Recently, the *NOTCH3* gene has been identified as a driver for tumor growth and resistance to radiation in meningiomas [15]. Choudhury et al. proposed that, due to its conserved expression in meningioma and potential to promote angiogenesis, *NOTCH3* is a potential therapeutic target for meningioma [15]. However, the metabolic consequences of *NOTCH3* activation in meningiomas remain poorly understood. Tumor metabolism is a key determinant of cancer cell survival and therapeutic response, yet meningiomas remain largely understudied in this context.

Through metabolomic and lipidomic profiling, this study demonstrates that *NOTCH3* activation influences lipid metabolism through the depletion of fatty acids, which are used by the tumor cells for FAO. This metabolic profile protects cells from ferroptosis and may be a mechanism to enhance malignancy, portending a poor prognosis in *NOTCH3-expressing* meningiomas.

## Materials and methods

### Cell culture

#### Meningioma cell lines and *NOTCH3* overexpression

The CH157-MN cell lines were obtained as a gift from Professor David Raleigh at UCSF and were cultured in DMEM (11960069, Life Technologies) supplemented with 10% FBS, 1× GlutaMAX (35050-061, Thermo Fisher Scientific), and 1× penicillin/streptomycin (15140122, Life Technologies). All cells were cultured at 37 °C and at 5% CO_2_ and 21% O_2_. Cell lines were confirmed to be mycoplasma-free at regular intervals.

The generation of *NOTCH3* ICD expressing meningioma cell lines was accomplished by creating the pLVX-Puro plasmid containing pCMV6-*NOTCH3*^ICD^ as previously described [15]. Lentiviral particles were produced by transfecting HEK293T cells with standard packaging vectors using the TransIT-Lenti Transfection Reagent (6605, Mirus). CH157-MN cells were stably transduced with lentiviral particles to generate *NOTCH3* ICD OE (CH157-MN^*NOTCH3* ICD^) or empty pLVX vector (CH157-MN^EV^) cells. Successfully transduced cells were isolated using Puromycin selection, and *NOTCH3* overexpression was confirmed using RT-qPCR.

### Patient samples

Human PDX samples were obtained under protocol #STU00095863 approved by the Northwestern Institutional Review Board (IRB). Meningioma tissue samples, PDX1 (WHO Grade 2) and PDX2 (WHO Grade 2), were obtained within one hour of surgical resection from 2 different patients and enzymatically dissociated in RPMI 1640 medium (Roswell Park Memorial Institute; Corning) containing 20 µL/mL collagenase (Roche) and 1 µL/mL DNase (Roche), supplemented with 2% fetal bovine serum (FBS). Single-cell suspensions were washed, seeded in culture plates, and expanded at 37 °C in Dulbecco’s Modified Eagle Medium (DMEM) supplemented with GlutaMAX™ (Gibco) and 10% FBS. *NOTCH3* expression was validated using RT-qPCR.

### Western blot

Cells were lysed in RIPA buffer (ThermoFisher, #89900) supplemented with protease inhibitors (ThermoFisher, #78430). Lysates were then centrifuged at 14,000 x g for 15 min at 4 °C, and the resulting supernatant was collected for protein quantification. Equal amounts of protein were mixed with 4x Laemmli sample buffer and boiled at 95 °C for 10 min to denature the proteins. Denatured samples were loaded onto SDS-PAGE gels and transferred to nitrocellulose membranes via wet transfer. Membranes were blocked with 5% non-fat milk in TBST for 1 h at room temperature, then incubated overnight at 4 °C with primary antibodies against NOTCH3 (Cell Signaling Technology, #2889, 1:500) and GAPDH (Cell Signaling Technology, #2118, 1:2000). After washing, the membrane was incubated with anti-rabbit IgG HRP-linked secondary antibody (Cell Signaling Technology, #7074, 1:4000) for 1 h at room temperature. Blots were developed using ECL reagent and visualized with a chemiluminescence imager.

### Flow cytometry analysis

PDX lines were lifted using trypsin and stained for viability using efluor780 fixable viability dye (Invitrogen 65-0865-14) 1:1000 in PBS on ice for 15 min. Anti-CD36 APC (Biolegend #336207) was subsequently added 1:100 to cells, washed and data acquired using a BD Symphony analyzer followed by analysis using Flow Jo software (Becton Dickinson).

### Real Time: quantitative PCR

RNA extraction was performed using either the Qiagen RNEasy kit (Qiagen, Hilden, Germany) or the Omega E.Z.N.A. RNA Isolation Kit (R6834-02, Omega Bio-Tek, Norcross, GA). The cDNA was generated from RNA samples with an iScript kit (BioRad, Hercules, CA, USA). Reactions were set up using standard amounts of cDNA, SybrGreen (Biorad), and forward and reverse primers (IDT, Newark, NJ, USA). The readout was performed on a CFX96 qPCR machine (Biorad). All primers were generated from Primer-BLAST using the fixed settings. Reactions were performed in triplicate. The following primers were used: *NOTCH3* (human) (forward 5’- GCCAAGCGGCTAAAGGTAGA-3’; reverse 5’- GGATGTCAGCAGCAACAAGA-3’), *CD36* (human) (forward 5’-CAGGTCAACCTATTGGTCAAGCC-3’; reverse 5’- GCCTTCTCATCACCAATGGTCC-3’), and β -actin for baseline expression level (human) (forward 5’- CACCATTGGCAATGAGCGGTTC-3’; reverse 5’- AGGTCTTTGCGGATGTCCACGT-3’). Fold changes in gene expression relative to untreated control were calculated by the ΔΔCt method using human actin as an endogenous control for mRNA expression.

### Untargeted metabolomic profiling

#### Metabolite extraction

CH157-MN^NOTCH*3* ICD^ and the CH157-MN^EV^cells were scraped and washed twice with PBS before pellets were flash-frozen and stored at − 80 °C until metabolite extraction. Pellets were resuspended in 80% methanol/20% H_2_O and then lysed by 3× cycles of heat shock (liquid nitrogen freezing followed by 42 °C water bath). Samples were then spun at 14,000 rpm for 15 min. The supernatants were collected and analyzed as described below.

#### Method for sample reconstitution after extraction

The extracted supernatants were dried using SpeedVac. 60% acetonitrile was added to the tube for reconstitution, followed by overtaxing for 30 s. The sample solution was then centrifuged for 30 min at 20,000 g, 4 °C. Samples were analyzed by High-Performance Liquid Chromatography and High-Resolution Mass Spectrometry and Tandem Mass Spectrometry (HPLC-MS/MS) as previously described [16].

### Bulk RNA sequencing

RNA was extracted using the RNeasy Plus Mini Kit (Qiagen, catalog no. 74134) according to the manufacturer’s protocol. RNA sample quality control, library preparation (polyA selection, nonstranded), sequencing of 20 M paired-end reads, and analysis were performed by Novogene.

### Human single cell sequencing

Single-cell RNA-seq data from 22 meningioma samples was extracted from publicly available repositories [17, 18] and processed according to prior descriptions in [18]. Briefly, the Seurat R Package using the scRNA-seq Seurat10x genomic workflow was used for all analyses unless noted otherwise [19]. Since data came from two different repositories, batch correction was performed using the standard Harmony algorithm [20]. Tumor cells were identified using CONICSmat [21] to determine copy-number loss of chromosome 22q using repetitions = 100 and postProb = 0.75. Cells with postProb < 0.15 were considered tumor and > 0.85 as normal. Clusters with more than 80% normal cells were considered non-meningioma clusters. .*NOTCH3* + meningioma cells were classified based on *NOTCH3* expression > 0. Seurat’s *FindMarkers* was used to find differentially expressed genes between *NOTCH3* + meningioma cells and *NOTCH3*- meningioma cells. Data was displayed using a volcano plot.

### Targeted fatty acid analyses

Cellular fatty acids were measured as previously described [22]. Briefly, cells were seeded at an initial density of 90,000 cells per well in a six-well plate in 2 ml of DMEM medium. A parallel plate of cells was scanned with an Incucyte Live-Cell Analysis System (Sartorius) and analyzed for confluence to normalize extraction buffer volumes based on cell number. An empty well was also extracted for a process control. After incubating cells for 24 h, lipids were extracted using an extraction buffer consisting of chloroform: methanol (containing 25 mg/L of butylated hydroxytoluene (Millipore Sigma, B1378)):0.88% KCl (w/v) at a final ratio of 8:4:3. The final extraction buffer also contained 0.75 µg/ml of norvaline and 0.7 µg/ml of cis-10-heptadecenoic acid as internal standards. For extraction, the medium was aspirated from cells, and cells were rapidly washed in ice-cold saline three times. The saline was aspirated, and methanol:0.88% KCl (w/v) (4:3 v/v) was added. Cells were scraped on ice, and the extract was transferred to 1.5 ml Eppendorf tubes (Dot Scientific, RN1700-GMT) before adding chloroform (Supelco, 1.02444). The resulting extracts were vortexed for 10 min and centrifuged at maximum speed (17000 x g) for 10 min. Lipids (organic fraction) were transferred to glass vials (Supelco, 29651-U) and dried under nitrogen gas for further analysis.

Fatty acids were analyzed as pentafluorobenzyl-fatty acid (PFB-FA) derivatives. Fatty acids were saponified from dried lipid pellets by adding 800 µl of 90% methanol/0.3 M KOH, vortexing, and incubating at 80 °C for 60 min. Each sample was then neutralized with 80 µl of formic acid (Supelco, FX0440). Fatty acids were extracted twice with 800 µl of hexane and dried under nitrogen gas. To derivatize, fatty acid pellets were incubated with 100 µl of 10% pentafluorobenzyl bromide (Sigma Aldrich, 90257) in acetonitrile and 100 µl of 10% N, N-diisopropylethylamine (Sigma Aldrich, D125806) in acetonitrile at room temperature for 30 min. PFB-FA derivatives were dried under nitrogen gas and resuspended in 50 µl of hexane for GC-MS analysis.

GC-MS was conducted with a TRACE TR-FAME column (ThermoFisher, 260M154P) installed in a Thermo Scientific TRACE 1600 gas chromatograph coupled to a Thermo ISQ 7610 mass spectrometer. Helium was used as the carrier gas at a constant flow of 1.8 ml/min. One microliter of sample was injected at 250 °C at a 4:1 split. After injection, the GC oven was held at 100 °C for 0.5 min, increased to 200 °C at 40 °C/min, held at 200 °C for 1 min, increased to 250 °C at 5 °C/min, and held at 250 °C for 11 min. The MS system operated under negative chemical ionization mode with methane gas at a flow rate of 1.25 ml/min, and the MS transfer line and ion source were held at 255 °C and 200 °C, respectively. The detector was used in scanning mode with an ion range of 150–500 m/z. Total ion counts were determined by integrating appropriate ion fragments for each PFB-FA using Skyline software [23, 24].

### Ferroptosis sensitivity and cell viability

CH157-MN^NOTCH3 ICD^ and CH157-MN^EV^ cells were plated at a density of 5 × 10^3^ cells/well in a flat-bottom, black 96-well plate treated with the designated amounts of RSL3 for 24 h as indicated within the figures. Cells were analyzed using the Cell Counting Kit-8 (CCK-8) (Sigma), and absorbance was measured at 450 nm using the Biotek Cytation 5 (Agilent). Cell viability was then normalized to untreated cells at each time point. Etomoxir, a CPT1a inhibitor, was utilized to inhibit mitochondrial function and FAO [25–27]; For assays with etomoxir, wells were pretreated with 5 µM of etomoxir for 4 h before treatment with the indicated concentrations of RSL3 for 24 h. Cells were again analyzed using CCK-8 as described above.

### Seahorse extracellular flux analysis and oxygen consumption rate (OCR)

The OCR was measured in a XF96 extracellular flux analyzer (Agilent Bioscience). The patient-derived meningioma cells, CH157-MN^NOTCH3 ICD^ and CH157-MN^EV^, were plated at a density of 2 × 10^4^ cells in 150 µL per well in a 96-well XF96 plate and allowed to adhere overnight. The cartridge from the Seahorse XFp FluxPak (Agilent; 103022-100) was hydrated overnight with XF Calibrant solution in a non-CO2 incubator. The plate was washed once with Agilent Seahorse XF Base Medium (Agilent; 103334-100) containing 2 mM glutamine (Agilent 103579-100) and 25 mM glucose prior to the addition of 150 µL Agilent Base Medium with treatment conditions of palmitate-BSA or BSA control. Palmitic acid, when added directly to cells, can induce lipotoxicity on its own. To avoid this, we utilized palmitate pre-conjugated to BSA in all assays (BSA-Palmitate Saturated Fatty Acid Complex (5 mM) at concentrations known to be appropriate for Seahorse assays [28, 29]. This approach minimizes toxicity and allows assessment of metabolic responses. After treatment with the above conditions, the plate was incubated for 40 min in a non-CO2 incubator before analysis. The Seahorse assay was then run with standard injections of the Seahorse XF Cell Mito Stress Test Kit (Agilent; 103015-100). Basal and maximal respiration rates were corrected by subtracting rotenone/antimycin A correction factor via previously reported methods [30, 31].

## Results

### *NOTCH3* + human meningioma cells upregulate CD36 expression

**Fig. 1:**
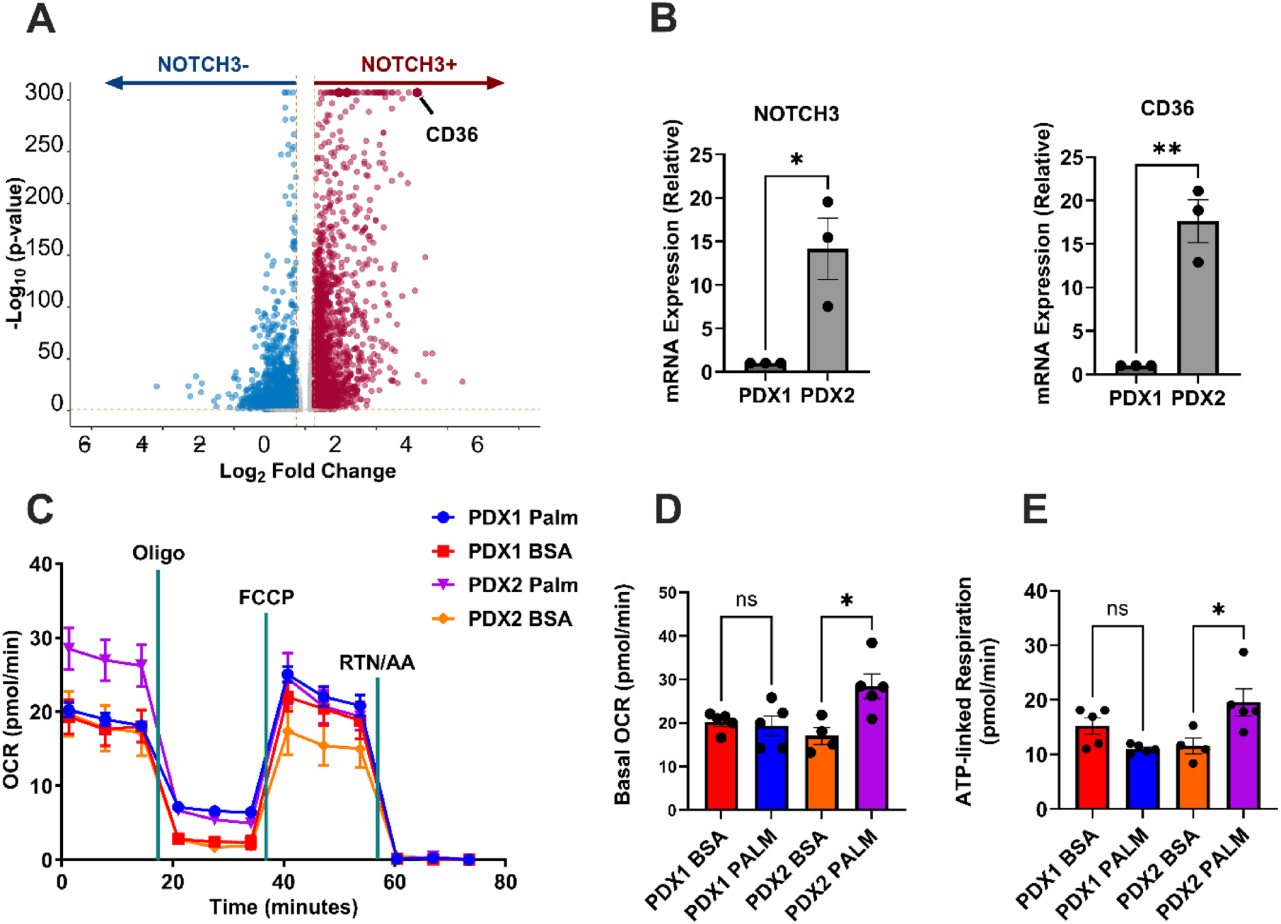
*NOTCH3* expression controls lipid uptake and oxidation in meningioma cells. **A.** Volcano plot of 14,080 *NOTCH3* + human meningioma cells from 22 meningiomas showing upregulation of CD36. P-values were calculated using DESeq2. **B.** Relative mRNA expression of NOTCH3 and CD36 in 2 patient-derived meningioma lines PDX1 and PDX2 (PDX1, *n* = 3; PDX2, *n* = 3). Experiments were performed in triplicate; significance was calculated using an unpaired Student’s t-test. **C.** Seahorse flux tracing of PDX1 and PDX2 under BSA or palmitate incubation conditions. **D**. Basal oxygen consumption rate (OCR) in PDX1 and PDX2 cells treated with palmitate-BSA or BSA control (PDX1, *n* = 3 in each condition; PDX2, *n* = 3 in each condition). **E**. ATP-linked respiration in PDX1 and PDX2 cells treated with palmitate-BSA or BSA control (PDX1, *n* = 3 in each condition; PDX2, *n* = 3 in each condition). In **C–E**, *n* = 2 independent experiments were performed. In **D–E**, a one-way ANOVA was performed with a Tukey’s post-hoc to calculate significance. *P* < 0.05*; *P* < 0.01**, *P* < 0.001***

To understand the transcriptional profile of *NOTCH3-expressing* meningiomas, 14,080 *NOTCH3* + human meningioma cells from publicly available data [17, 18] were analyzed. The scRNA-seq data revealed a correlation between *NOTCH3* + meningioma cells and clusters-of-differentiation 36 (*CD36*) expression, indicating that *CD36* is enriched in *NOTCH3* + cells (Fig. 1A). The lipid metabolism enrichment observed in *NOTCH3*-expressing patient-derived meningioma cells led us to investigate the direct role of *NOTCH3* overexpression using a controlled in vitro model.

### *NOTCH3* + primary meningioma cells exhibit increased lipid metabolism

To further investigate the consequences of *NOTCH3* expression on lipid metabolism in human meningioma cells, two patient-derived meningioma lines were profiled for *NOTCH3* expression via RT-qPCR. PDX1 exhibited low and PDX2 exhibited high *NOTCH3* expression (Fig. 1B). The expression of CD36 was higher in PDX2 than in PDX1 (Fig. 1B), consistent with the scRNA-seq data analysis. Since CD36 is associated with the uptake of saturated fatty acids for FAO,^2^ we hypothesized that the *NOTCH3*^high^ PDX2 would be more proficient at FAO than *NOTCH3*^low^ PDX1. In addition to qPCR, we also validated increased CD36 and NOTCH3 expression using flow cytometry for CD36 (Supplementary Fig. 1A-C), and western blot for NOTCH3, respectively (Supplementary Fig. 1D). To test this, Seahorse extracellular flux analysis was performed on both PDX lines with the inclusion of a FA palmitate-BSA conjugate, or a BSA alone control (Fig. 1C–E). In *NOTCH3*^lo^ PDX1, there was no difference in basal OCR with the addition of palmitate; whereas PDX2 (*NOTCH3*^high^) responded with a significantly higher basal OCR with palmitate (17.0 ± 1.9 pmol O_2_/min in BSA incubated vs. 28.5 ± 3.2 in the palmitate-treated group; *p* < 0.05) (Fig. 1D). Furthermore, ATP-linked respiration was also increased in the *NOTCH3*^high^ PDX2, whereas no change occurred in the *NOTCH3*^low^ PDX1 (Fig. 1E). This data suggests that *NOTCH3* controls lipid metabolism in meningioma cells and prompted the use of an overexpression line to verify that the increase in FAO is directly related to *NOTCH3* activity.

### *NOTCH3* overexpression alters lipidomic profile

**Fig. 2:**
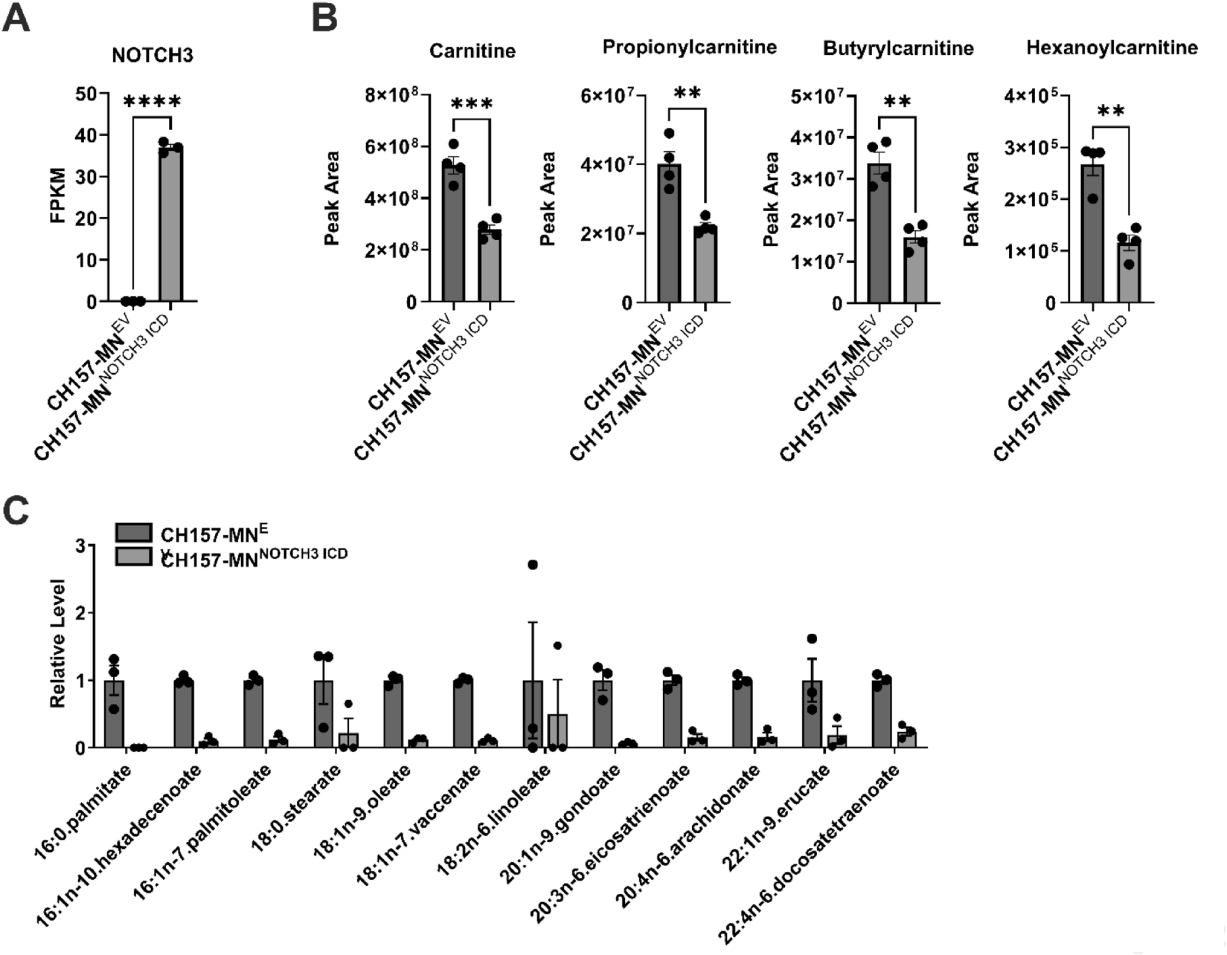
*NOTCH3* overexpression alters fatty acid composition in meningioma cells. **A** Bulk RNA sequencing FPKM values of *NOTCH3* in CH157-MN^EV^ and CH157-MN^NOTCH3 ICD^ (CH157-MN^EV^, *n* = 3; CH157-MN^NOTCH3 ICD^, *n* = 3). **B** Untargeted metabolomic analysis showing a decrease in acyl carnitine species carnitine, propionylcarnitine, butyrylcarnitine, and hexanoylcarnitine in CH157-MN^NOTCH3 ICD^ compared with CH157-MN^EV^ (CH157-MN^EV^, *n* = 4; CH157-MN^NOTCH3 ICD^, *n* = 4). Significance was calculated using unpaired Student’s t-tests. *P* < 0.05*; *P* < 0.01**, *P* < 0.001***; *P* < 0.0001****. **C** Histogram of targeted lipidomic analysis of CH157-MN^EV^ and CH157-MN^NOTCH3 ICD^ (CH157-MN^EV^, *n* = 3; CH157-MN^NOTCH3 ICD^, *n* = 3)

To isolate and directly study the role of *NOTCH3* and its effect on metabolism in meningiomas, the CH157-MN^NOTCH3 ICD^ and CH157-MN^EV^ cells were used for further in vitro studies (Fig. 2A). Metabolomic profiling was performed on CH157-MN^NOTCH3 ICD^ and CH157-MN^EV^ cells to determine the metabolite differences between the two cell lines. Compared to EV control, CH157-MN^NOTCH3 ICD^ exhibits decreases in several fatty acyl carnitine species required for fatty acid transport into the mitochondria for FAO (Fig. 2B). This was further validated with targeted fatty acid profiling that confirmed decreased saturated and unsaturated fatty acid levels in CH157-MN^NOTCH3 ICD^ compared with the EV control (Fig. 2C).

### *NOTCH3* controls transcriptional expression of lipid metabolic genes

**Fig. 3:**
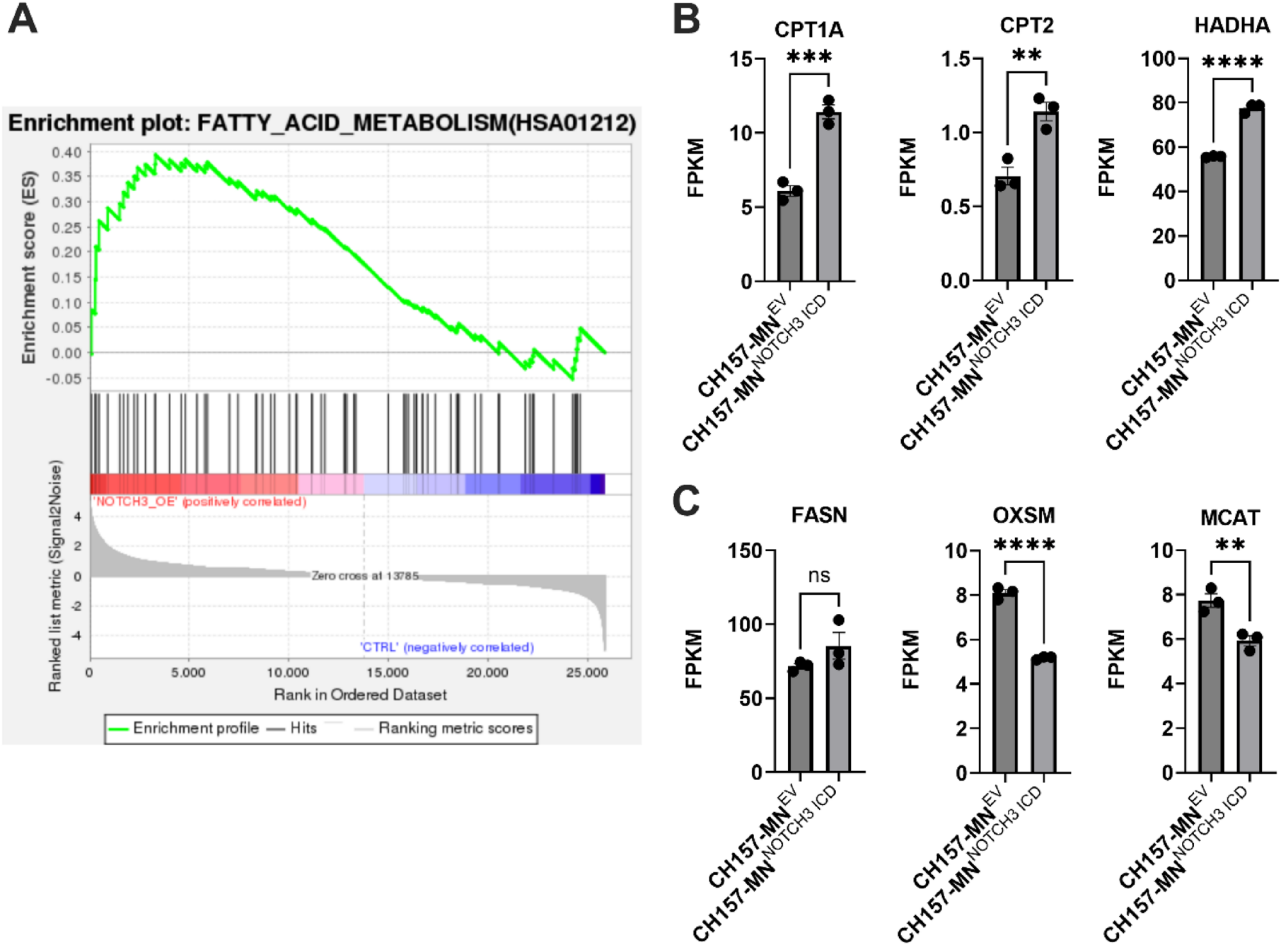
*NOTCH3* overexpression enhances transcription of fatty acid oxidation genes in meningioma cells. **A** Gene set enrichment analysis (GSEA) of bulk RNA sequencing showing increased activation of fatty acid metabolism pathways in CH157-MN^NOTCH3 ICD^ compared with CH157-MN^EV^ (CH157-MN^EV^, *n* = 3; CH157-MN^NOTCH3 ICD^, *n* = 3). **B** Bulk RNA sequencing FPKM values of CH157-MN^EV^ and CH157-MN^NOTCH3 ICD^ showing upregulation of FAO genes CPT1A, CPT2, and HADHA (CH157-MN^EV^, *n* = 3; CH157-MN^NOTCH3 ICD^, *n* = 3). **C** Bulk RNA sequencing FPKM values of CH157-MN^EV^ and CH157-MN^NOTCH3 ICD^ showing no difference or decrease in fatty acid synthesis genes FASN, OXSM, and MCAT (CH157-MN^EV^, *n* = 3; CH157-MN^NOTCH3 ICD^, *n* = 3). In **B–C**, significance was calculated using unpaired Student’s t-tests. *P* < 0.05*; *P* < 0.01**, *P* < 0.001***; *P* < 0.0001****

RNA sequencing of CH157-MN^NOTCH3 ICD^ and CH157-MN^EV^ cell lines showed increased enrichment of fatty acid metabolism genes in CH157-MN^NOTCH3 ICD^ cells (Fig. 3A). Specifically, key FAO genes such as CPT1A and CPT2, which import long-chain fatty acids into mitochondria for oxidation, and HADHA, which encodes for a mitochondrial enzyme that is essential for β-oxidation, were upregulated in CH157-MN^NOTCH3 ICD^ cells (Fig. 3B). In contrast, genes essential to fatty acid biosynthesis/anabolism, including FASN (fatty acid synthase), OXSM (3-oxoacyl-ACP synthase), and MCAT (malonyl-CoA acyltransferase), were downregulated or unchanged (Fig. 3C). These data suggest that *NOTCH3* promotes a shift from fatty acid synthesis to FAO in meningioma.

### *NOTCH3* overexpression increases maximal oxidative respiration in the presence of fatty acids

**Fig. 4:**
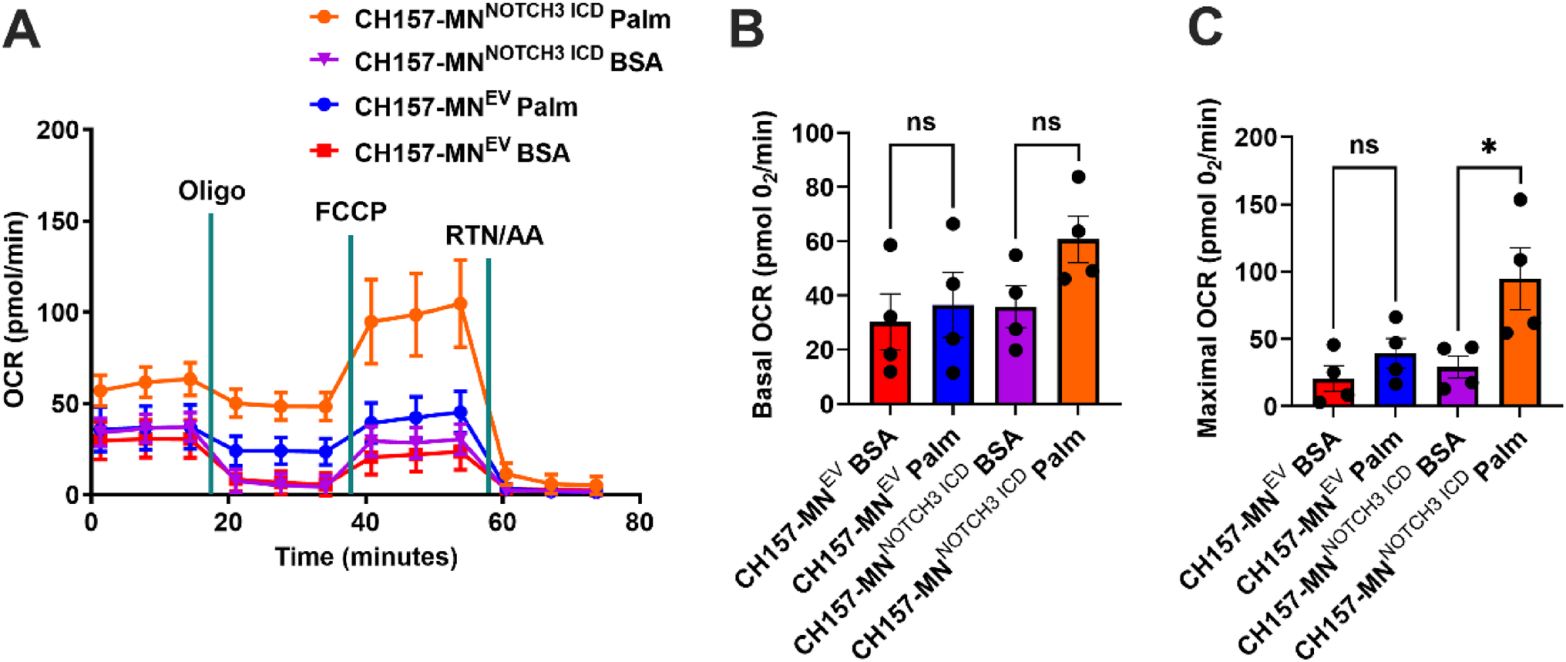
*NOTCH3* overexpression increases maximal oxidative respiration in the presence of lipids. **A** Oxygen consumption rate (OCR) of CH157-MN^EV^ and CH157-MN^NOTCH3 ICD^ treated with palmitate-BSA or BSA control (CH157-MN^EV^, *n* = 4 in each condition; CH157-MN^NOTCH3 ICD^, *n* = 4 in each condition). **B** Basal corrected oxygen consumption rate (OCR) in CH157-MN^EV^ and CH157-MN^NOTCH3 ICD^ cells treated with palmitate-BSA or BSA control (CH157-MN^EV^, *n* = 4 in each condition; CH157-MN^NOTCH3 ICD^, *n* = 4 in each condition). C Maximal corrected oxygen consumption rate (OCR) in CH157-MN^EV^ and CH157-MN^NOTCH3 ICD^ cells treated with palmitate-BSA or BSA control (CH157-MN^EV^, *n* = 4 in each condition; CH157-MN^NOTCH3 ICD^, *n* = 4 in each condition). In **B–C**, a one-way ANOVA was performed with a Tukey’s post-hoc to calculate significance. *P* < 0.05*; *P* < 0.01**, *P* < 0.001***

To understand the functional metabolic impact of *NOTCH3* expression in meningioma cells, the OCR of CH157-MN^NOTCH3 ICD^ versus CH157-MN^EV^ was compared using the Seahorse extracellular flux assay in the presence of palmitate and BSA palmitate control (Fig. 4A). As in the PDX experiments, the presence of palmitate trended towards an increase in basal OCR only in *NOTCH3* overexpressing lines (*p* = 0.06, Fig. 4B). Unlike the PDX lines, FCCP (FCCP is a mitochondrial uncoupler that increases maximal respiration by collapsing the proton gradient, forcing cells to operate at their maximum respiratory capacity) increased the maximal respiration after the injection (29.3 ± 8.1 pmol O_2_/min in BSA incubated vs. 94.8 ± 23.0 in the palmitate-treated group; *p* < 0.05) (Fig. 4C). This data supports the hypothesis that *NOTCH3* upregulates FAO in meningioma.

### *NOTCH3* overexpression confers resistance to ferroptosis

**Fig. 5:**
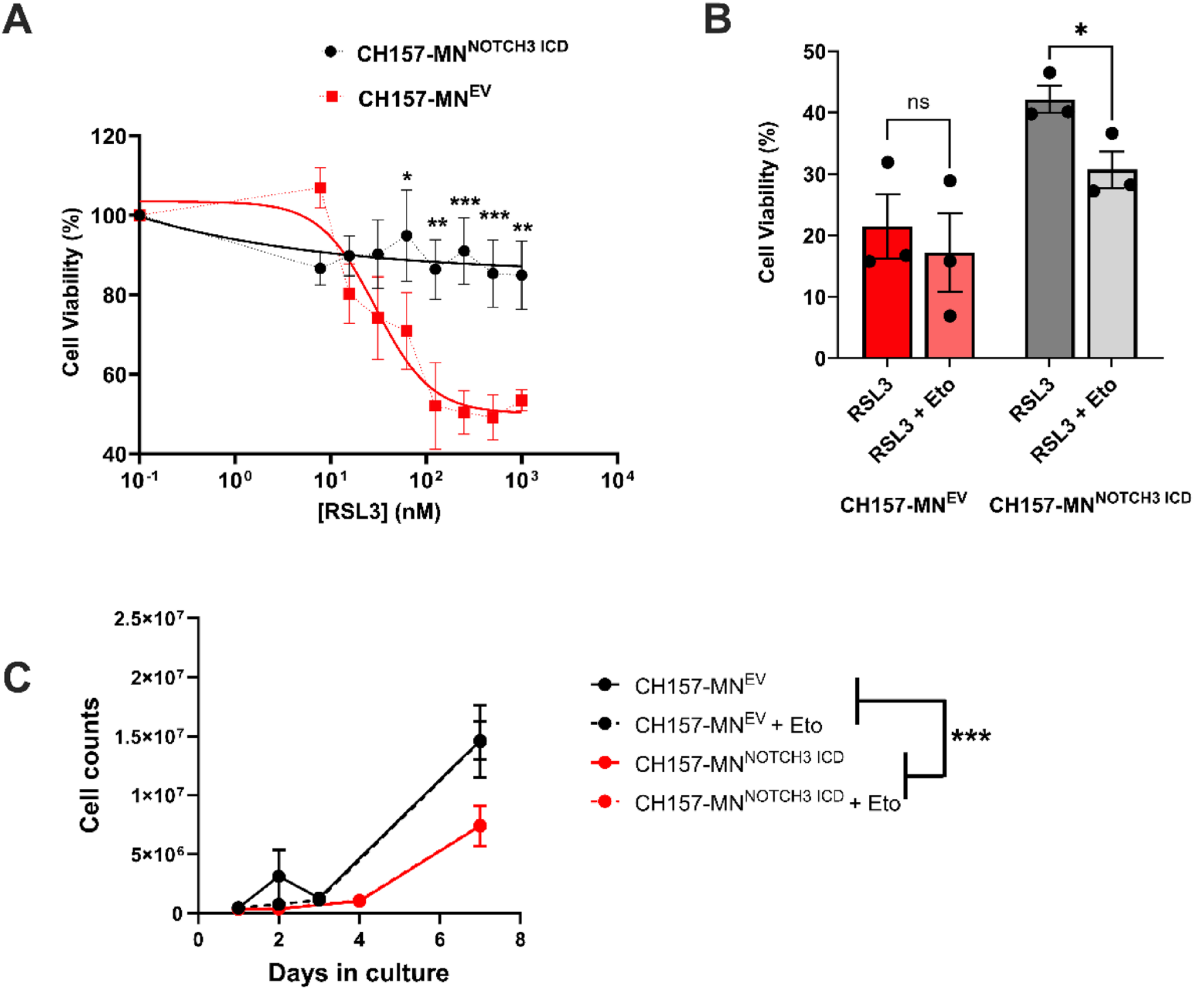
*NOTCH3*-driven lipid depletion confers resistance to ferroptosis. **A** Cell viability of CH157-MN^EV^ and CH157-MN^NOTCH3 ICD^ treated with increasing concentration of RSL3 for 24 h (CH157-MN^EV^, *n* = 5; CH157-MN^NOTCH3 ICD^, *n* = 5). To test for significance in curves, a two-way ANOVA was performed, and Sidak’s multiple comparison post hoc test was used to test for individual significance across rows. **B** Cell viability of CH157-MN^EV^ and CH157-MN^NOTCH3 ICD^ treated with or without 5 µM of etomoxir with 32.5 nM RSL3 (CH157-MN^EV^, *n* = 3 in each condition; CH157-MN^NOTCH3 ICD^, *n* = 3 in each condition). **C**, Proliferation of CH157-MN^EV^ and CH157-MN^NOTCH3 ICD^ over several days with the inclusion of 5 µM etomoxir. A one-way ANOVA was performed with a Tukey’s post-hoc to calculate significance in **B**. In **A** and **C**, Two-way ANOVA, followed by a Tukey’s post-hoc was used for individual comparison. In (C), *P* < 0.05*; *P* < 0.01**, *P* < 0.001***

To understand the impact of increased FAO in CH157-MN^NOTCH3 ICD^ cells on ferroptosis, a series of cell death experiments utilizing RSL3, a ferroptosis inducer, was conducted. Utilizing RSL3, which inhibits glutathione peroxidase 4 (GPX4) and induces ferroptosis, the percent viability of CH157-MN^NOTCH3 ICD^ was compared with EV cells. At increasing concentrations of RSL3, CH157-MN^NOTCH3 ICD^ cells exhibit complete resistance to ferroptosis induction when compared with CH157-MN^EV^ (Fig. 5A). Considering the protection from RSL3 observed in the CH157-MN^NOTCH3 ICD^, and the low levels of intracellular lipids in this line, we hypothesized *NOTCH3* protects meningioma cells from ferroptosis via their increased FAO. To test this hypothesis, we treated CH157-MN^EV^ and CH157-MN^NOTCH3 ICD^ cells with 5 µM of etomoxir (a FAO inhibitor) co-incubated with 32.5 nM RSL3 for 24 h (Fig. 5B). While the addition of etomoxir did not potentiate the cell death of CH157-MN^EV^, there was a significant decrease in viability in the CH157-MN^NOTCH3 ICD^ (42.2 ± 2.2% in RSL3 compared to 30.7 ± 3.0% in RSL3 with Eto; *p* < 0.05), demonstrating that enhanced FAO is protective against ferroptosis in meningioma cells. To determine whether increased proliferation underlies the observed resistance to RSL3, we performed proliferation assays comparing CH157-MN^NOTCH3 ICD^ and CH157-MN^EV^. Contrary to expectations, CH157-MN^NOTCH3 ICD^ proliferated more slowly than vector control cells (Fig. 5C). These data suggest that resistance to ferroptosis is unlikely due to increased proliferative capacity. To further evaluate whether fatty acid oxidation is required for meningioma cell survival independently of RSL3, we treated cells with 5 µM etomoxir alone. Treatment with etomoxir did not significantly affect the viability of either CH157-MN^EV^ or CH157-MN^NOTCH3 ICD^ cells (Fig. 5C). These data suggest that NOTCH3 expression may increase reliance on fatty acid oxidation for maintaining redox homeostasis, thereby creating a vulnerability to ferroptotic stress when fatty acid oxidation is inhibited.

## Discussion

In this study, we demonstrate that *NOTCH3* promotes a metabolic shift towards FAO, lipid depletion, and resistance to ferroptosis in meningioma cells. In previous studies, *NOTCH3* expression has been associated with higher grade, radiation resistance, and recurrence in meningioma [15]. However, the metabolic role of *NOTCH3* in meningiomas has not been established. In multiple tumor types, alterations in lipid metabolism have contributed to treatment resistance and malignancy [32–35]. This study is the first to identify an association between *NOTCH3* expression, FAO regulation, and ferroptosis resistance in meningioma cells.

Through scRNA-seq, we demonstrate that *NOTCH3* + human meningioma cells exhibit enriched expression of *CD36*. CD36 is a membrane-bound fatty acid transporter that facilitates the uptake of long-chain fatty acids (LCFAs), directing them toward FAO [35, 36]. CD36-driven FAO enhances tumor cell survival in stress-induced environments by providing an alternative energy source, reducing reactive oxygen species (ROS)-mediated cell death, and maintaining cancer stem cell (CSC) populations, contributing to chemotherapy, radiotherapy, and immunotherapy resistance [37–40]. CD36 has been shown to facilitate evasion of therapy-induced metabolic stress in tumor cells [40, 41]. We established *NOTCH3* high and low patient-derived primary meningioma lines and validated that *NOTCH3* correlates with *CD36* expression. By comparing OCR between the *NOTCH3*^high^ PDX2 and *NOTCH3*^low^ PDX1 primary lines, we show an increase in maximal mitochondrial respiration in the presence of palmitate in *NOTCH3*^high^ primary cells, suggesting a functional upregulation of lipid metabolism in *NOTCH3*-expressing meningioma cells.

Utilizing the CH157-MN meningioma model, we demonstrate a shift in lipid metabolism with *NOTCH3* ICD overexpression. Untargeted metabolomic profiling of CH157-MN^NOTCH3 ICD^ revealed decreased fatty acyl carnitine species when compared with CH157-MN^EV^ controls. Upon bulk RNA sequencing, CH157-MN^NOTCH3 ICD^ cells exhibit upregulation of canonical FAO and simultaneous downregulation of FAS genes, confirming enhancement of lipid metabolism with *NOTCH3* overexpression [42–45]. Complementary targeted fatty acid analyses revealed depletion of intracellular fatty acids in CH157-MN^NOTCH3 ICD^ cells. Functionally, extracellular flux assays demonstrate elevated maximal mitochondrial respiration in the presence of palmitate, confirming increased mitochondrial oxidative capacity in CH157-MN^NOTCH3 ICD^ cells.

Lipid bioavailability is critical to ferroptosis, a non-apoptotic form of cell death caused by toxic iron accumulation and lipid peroxidation [46–48]. Through the generation of reactive oxygen species (ROS), ferroptosis acts as a tumor suppressor mechanism [48]. This study demonstrates reduced sensitivity to RSL3, a ferroptosis inducer, in CH157-MN^NOTCH3 ICD^ cells. Co-treatment with etomoxir, an inhibitor of CPT1-dependent mitochondrial fatty acid import, restored RSL3-induced ferroptosis in CH157-MN^NOTCH3 ICD^ cells. In a similar study, investigators report *NOTCH3* expression negatively regulates ROS-mediated lipid peroxidation in non-small cell lung carcinoma (NSCLC), increasing tumorigenesis [49]. Importantly, CH157-MN^NOTCH3 ICD^ cells do not proliferate faster than CH157-MN^EV^ cells, suggesting RSL3 resistance is not due to increased growth. Their slightly reduced proliferation may even limit sensitivity to lipid peroxidation. While etomoxir alone had no effect, its combination with RSL3 reduced viability, supporting a model where FAO is dispensable at baseline but essential for redox balance under ferroptotic stress. These results suggest that *NOTCH3*-driven lipid depletion protects cells from ferroptosis, which may confer a survival advantage in meningioma.

These findings suggest that *NOTCH3* is a central mediator of fatty acid metabolism in meningiomas. By upregulating FAO pathways, *NOTCH3*-expressing meningioma cells may enhance ATP generation while reducing susceptibility to ferroptotic death processes. The combined benefit of improved fatty acid utilization and cell survival may confer an advantage under conditions of metabolic or oxidative stress, particularly in the context of radiation. These data serve as one potential mechanism of why *NOTCH3* expression portends a poor prognosis, as *NOTCH3* + meningiomas are higher grade, more likely to recur, and exhibit resistance to radiation therapy [15]. FAO, with adjuvant CPT1a inhibitors, may restore ferroptosis vulnerability in *NOTCH3*-expressing meningiomas. This approach could be efficacious when combined with ferroptosis inducers or radiation therapy, both of which rely on oxidative stress as a mechanism of cytotoxicity.

Our findings suggest that *NOTCH3* expression does not simply enhance the capacity for fatty acid oxidation in parallel with other metabolic pathways but rather shifts cellular metabolism toward a greater dependency on fatty acid oxidation. This metabolic reprogramming may contribute to maintaining redox balance, making *NOTCH3*-expressing meningioma cells more susceptible to ferroptosis when fatty acid oxidation is blocked. The observed synergy between etomoxir and RSL3 in these cells highlights fatty acid oxidation as a potential metabolic liability in the context of *NOTCH3* activation. While the precise mechanism underlying this selective sensitivity remains to be elucidated, it may reflect an increased need for fatty acid-derived substrates to support antioxidant defenses in *NOTCH3*-expressing meningioma cells.

This study has several limitations. All experiments were performed in an in vitro setting using primary and established meningioma cell lines. While this study focuses on a mechanistic investigation, further in vivo studies are needed to assess the role of *NOTCH3* in meningioma metabolism. An important limitation of our study is that we did not directly assess lipid flux or carnitine utilization dynamics in *NOTCH3*-expressing cells. Future studies using isotope tracing or other flux analyses will be critical to determine whether the observed lipid depletion and changes in carnitine species reflect increased fatty acid uptake and rapid mitochondrial catabolism. Additionally, this study did not assess clinical outcomes in patient samples stratified by *NOTCH3* expression, as this was outside the scope of this investigation. Future studies should incorporate transcriptomic and metabolomic profiling of patient-derived tumors stratified by WHO grade to validate the clinical implications of our findings.

Our study identifies a role for *NOTCH3* in promoting fatty acid oxidation in meningioma cells. However, we acknowledge that the precise mechanisms by which *NOTCH3* drives this metabolic reprogramming remain to be defined. Future work will focus on dissecting how *NOTCH3* signaling modulates key regulators of lipid metabolism, including potential direct and indirect effects on fatty acid transport, oxidation enzymes, and associated transcriptional programs.

## Conclusions

These findings highlight the role of *NOTCH3* in mediating FAO in meningiomas. Increased utilization of lipids in *NOTCH3*-expressing meningiomas poses a metabolic advantage as well as contributes to resistance to ferroptosis, leading to increased cell survival in the setting of oxidative stress. These data highlight a potential mechanism for increased malignant potential of *NOTCH3* + meningiomas.

## Supplementary Information

Below is the link to the electronic supplementary material.

Supplementary Material 1

## Data Availability

Any data is available at the request of the corresponding author, Jason Miska (Jason.miska@northwestern.edu). RNA-sequencing data can be accessed via BioProject accession number PRJNA1124134 (https://www.ncbi.nlm.nih.gov/bioproject/1124134).
