# Supplementary material for: NOTCH3 drives fatty acid oxidation and ferroptosis resistance in aggressive meningiomas
Source: J Neurooncol. 2025 Sep 9;175(3):979–91. doi: 10.1007/s11060-025-05208-5 (PMC12511256; doi:10.1007/s11060-025-05208-5)
Supplement: Supplementary file 1 — Supplementary Material 1 [file 11060_2025_5208_MOESM1_ESM.docx]

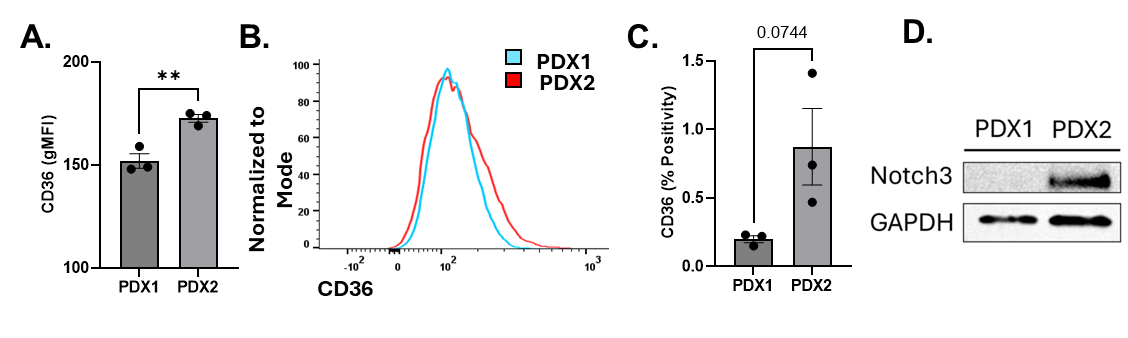


**Supplementary Figure 1. Protein expression of CD36 in meningioma patient-derived xenografts.** In **A-C**, PDX1 (NOTCH3^lo^) and PDX2 (NOTCH3^hi^) was analyzed for CD36 using antibody staining and flow cytometric analysis. In **D**, validation of Notch3 expression using western blot analysis.
